# Glomerular endothelial glycocalyx-derived heparan sulfate inhibits glomerular leukocyte influx and attenuates experimental glomerulonephritis

**DOI:** 10.3389/fmolb.2023.1177560

**Published:** 2023-06-01

**Authors:** Marissa L. Maciej-Hulme, Jasper J. Van Gemst, Patience Sanderson, Angelique L. W. M. M. Rops, Jo H. Berden, Bart Smeets, I. Jonathan Amster, Ton J. Rabelink, Johan Van Der Vlag

**Affiliations:** ^1^ Department of Nephrology, Radboud Institute for Molecular Life Sciences, Radboud University Medical Center, Nijmegen, Netherlands; ^2^ Department of Chemistry, University of Georgia, Athens, GA, United States; ^3^ Department of Pathology, Radboud Institute for Molecular Life Sciences, Radboud University Medical Center, Nijmegen, Netherlands; ^4^ Department of Nephrology, Einthoven Laboratory for Vascular Medicine, Leiden University Medical Center, Leiden, Netherlands

**Keywords:** heparan sulfate, glomerulonephritis, leukocyte, glomerular endothelial cell, inflammation, glycocalyx

## Abstract

Proliferative forms of glomerulonephritis are characterized by the influx of leukocytes, albuminuria, and loss of kidney function. The glomerular endothelial glycocalyx is a thick carbohydrate layer that covers the endothelium and is comprised of heparan sulfate (HS), which plays a pivotal role in glomerular inflammation by facilitating endothelial-leukocyte trafficking. We hypothesize that the exogenous glomerular glycocalyx may reduce the glomerular influx of inflammatory cells during glomerulonephritis. Indeed, administration of mouse glomerular endothelial cell (mGEnC)-derived glycocalyx constituents, or the low-molecular-weight heparin enoxaparin, reduced proteinuria in mice with experimental glomerulonephritis. Glomerular influx of granulocytes and macrophages, as well as glomerular fibrin deposition, was reduced by the administration of mGEnC-derived glycocalyx constituents, thereby explaining the improved clinical outcome. HS_glx_ also inhibited granulocyte adhesion to human glomerular endothelial cells *in vitro*. Notably, a specific HS_glx_ fraction inhibited both CD11b and L-selectin binding to activated mGEnCs. Mass spectrometry analysis of this specific fraction revealed six HS oligosaccharides, ranging from tetra- to hexasaccharides with 2–7 sulfates. In summary, we demonstrate that exogenous HS_glx_ reduces albuminuria during glomerulonephritis, which is possibly mediated via multiple mechanisms. Our results justify the further development of structurally defined HS-based therapeutics for patients with (acute) inflammatory glomerular diseases, which may be applicable to non-renal inflammatory diseases as well.

## 1 Introduction

Glomerulonephritis and other inflammatory glomerular diseases are characterized by renal injury and loss of kidney function. Glomerular inflammation involves the interaction of cytokines, chemokines, complement proteins, leukocytes, and the glomerular endothelial glycocalyx (Butcher, 1991; Elhadj et al., 2002; Rops et al., 2004a; Parish, 2006; Taylor and Gallo, 2006; Rops et al., 2008). The endothelial glycocalyx is a thick carbohydrate layer rich in glycosaminoglycans (GAGs), including chondroitin sulfate (CS) and heparan sulfate (HS) (Esko and Selleck, 2002; Reitsma et al., 2007; Gao and Lipowsky, 2010). In particular, HS mediates several inflammatory processes. HS is synthesized as a proteoglycan side chain and consists of repeating β1-4- and α1-4-linked N-acetylglucosamine (GlcNAc) and glucuronic acid (GlcA) disaccharides. During its synthesis, the backbone of HS is extensively modified by various sulfotransferases and an epimerase, which leads to C-5 epimerization and N-, 2-O, 3-O, and 6-O sulfation, thereby creating structural heterogeneity within the glycan and a distinct domain structure (Esko and Selleck, 2002; Morimoto-Tomita et al., 2002; Xia et al., 2002). The expression of HS-modifying enzymes in response to certain (proinflammatory) stimuli is differentially regulated in different tissues and cell types (Bennett et al., 1995; Marr et al., 1997; Wong et al., 2000; Iozzo, 2001; Saphire et al., 2001; Rops et al., 2004a; Iozzo, 2005), resulting in tissue- and cell-dependent expression of specific HS domains (Jenniskens et al., 2000; Dennissen et al., 2002). Due to its immense structural diversity, HS is the key GAG involved in multiple inflammatory processes through binding of cytokines, chemokines, and leukocyte adhesion molecules, such as L-selectin and CD11b/macrophage-1 antigen 4 (Diamond et al., 1995; Wang et al., 2005). Various studies have described the importance of HS for the interaction of leukocytes with endothelium (Diamond et al., 1995; Wang et al., 2002; Celie et al., 2005; Parish, 2005; Wang et al., 2005; Rops et al., 2008; Rops et al., 2014). Leukocyte trafficking is characterized by several stages: tethering, rolling, firm adhesion, and extravasation of the leukocyte through the endothelium into the tissue (Butcher, 1991; Schlondorff et al., 1997), with HS playing a prominent role in each of these steps (Handel et al., 2005; Parish, 2006). By using different *in vitro* and *in vivo* approaches, we previously showed that specific HS domains in the glomerular endothelial glycocalyx are involved in binding of leukocytes and chemokines (Rops et al., 2004b; Rops et al., 2007a; Rops et al., 2008; Rops et al., 2014; van Gemst et al., 2018). Both healthy and activated cultured glomerular endothelial cells (mGEnCs), as well as *in vivo* on glomerular endothelium in experimental and human glomerular diseases, express these specific HS domains involved in glomerular inflammation, with increased expression prominent during inflammation (Rops et al., 2008; van Gemst et al., 2018). Therefore, we hypothesized that isolated glycocalyx and HS isolated from the glycocalyx of glomerular endothelial cells have the potential to inhibit glomerular leukocyte influx and/or adhesion to glomerular endothelium, thus dampening the inflammatory response and improving disease outcome. Our collective results reveal that specific glomerular endothelial glycocalyx-derived HS fractions affect the interaction between inflammatory cells and the glomerular endothelium, thereby leading to a better outcome in experimental glomerulonephritis.

## 2 Materials and methods

### 2.1 Cell culture

Conditionally immortalized mouse glomerular endothelial cells (mGEnCs) with all features of primary mouse glomerular endothelial cells were cultured as previously described (Rops et al., 2004b). Briefly, for experiments, cells were cultured in differentiation media (DMEM:HAM-F12 3:1, Invitrogen Life Technologies) supplemented with 5% FBS (Biochrom SO113/115 batch 0667B) and 1% penicillin/streptomycin (Gibco) at 37°C/5% CO_2_ for 7°days prior to treatment. Murine leukocyte 32Dcl3 cells were cultured in Roswell Park Memorial Institute (RPMI) 1640 medium (Dutch modification, Gibco) containing 10% FBS (Bodinco), 1% penicillin/streptomycin, 1% glutamate (Gibco), 1% pyruvate (Gibco), and 1 ng/mL IL-3 (PeproTech). The culture was maintained in 5% CO_2_ at 37°C with concentration readjustment to 5×10^5^ cells/mL. Human renal glomerular endothelial cells (HRGECs, ScienCell) were cultured in fibronectin (PromoCell) pre-coated tissue culture plasticware (COSTAR) between passages 2–5 with endothelial cell medium (ScienCell, #1001) supplemented with 5% FBS (ScienCell, #0025), 1% endothelial cell growth supplement (ScienCell, #1052), and 1% penicillin/streptomycin (ScienCell, #0503) in 5% CO_2_ at 37°C. Cells were passaged using 0.05% trypsin/0.5 mM EDTA solution (ScienCell, #0183) and trypsin neutralization solution (ScienCell, #0113). Cells were seeded onto new fibronectin pre-coated tissue culture plasticware at a density of 5,000 cells/cm^2^, and the medium was replaced every 48 h until 95% confluency. Where indicated, glomerular endothelial cells were treated with 10 ng/mL mouse recombinant TNFα (PeproTech) or 1 μg/mL LPS *O111:B4* from *E. coli* (Sigma) for 18 h.

### 2.2 Extraction, isolation, and fractionation of glycocalyx constituents

Glycocalyx was extracted from unstimulated cell layers by overnight digestion with 125 μg/mL proteinase K (Merck Chemicals B.V., Amsterdam, Netherlands) in 50 mM Tris-HCl (pH: 7.9), 10 mM NaCl, 3 mM MgCl_2_, and 1% triton X-100 buffer, followed by overnight DNAse-I (QIAGEN) and RNAse (GE Healthcare) treatment at 37°C. NaCl was added to digested extracts (final concentration of 2 M), followed by chloroform (1:1), vortexing, and centrifugation for 20 min at ×4,636 g to separate the phases. The upper layer (aqueous phase) was dialyzed against 5 × 5 L baths of Milli-Q H_2_O using SnakeSkin dialysis membranes (MWCO 3500 Da, Thermo Scientific) and dried using a Savant SC210A SpeedVac concentrator (Thermo Scientific). To isolate the individual GAG constituents, mGEnC glycocalyx was separated on 1% agarose gel in barium acetate (van de Lest et al., 1994; van Gemst et al., 2016), followed by excision and phenol extraction of the separated HS and CS. Individual fractions were ethanol-extracted several times to remove phenolic contamination. Isolated GAGs were analyzed by barium acetate agarose gel electrophoresis for determination of GAG concentration and purity, as described previously (van de Lest et al., 1994; van Gemst et al., 2016). Size fractionation of purified mGEnC HS_glx_ was performed in 0.25 M ammonium bicarbonate at 0.22 mL/min using a BioGel P10 resin column (75 × 16 mm, 90–180 µm beads, Bio-Rad). Then, 1 mL fractions were collected and pooled into corresponding peaks (Supplementary Figure S7). Pooled fractions were dialyzed against Milli-Q water and dried. To further reduce the size, F1 was digested with heparinase III (1 IU/mL, Iduron) in 0.1 M sodium acetate and 0.1 mM calcium acetate pH 7.0 buffer at 37°C for 18 h, heated to 95°C for 10 min to inactivate the enzyme, and then buffer-exchanged with Milli-Q water and dried. Isolation of unstimulated HRGEC HS_glx_ from extracted glycocalyx was performed as described previously (Guimond et al., 2009; Maciej-Hulme et al., 2023). In brief, dialyzed and concentrated HRGEC glycocalyx extracts were digested with 125 mU of chondroitinase ABC (Sigma) in 25 mM Tris and 2 mM Mg(Ac)_2_ pH 8 for 18 h before fractionation by anion exchange chromatography using DEAE-Sepharose CL-6B beads (Sigma) equilibrated in PBS. Bound HS_glx_ was washed with 0.25 M NaCl in PBS, pH 7.4 and then eluted with 2 M NaCl in PBS, pH 7.4. Isolated HS_glx_ was desalted via PD10 desalting columns (GE Healthcare, Sephadex G25) using Milli-Q H_2_O. Resultant HS_glx_ was dried before resuspension in sterile Milli-Q H_2_O (1 μL/cm^2^ of original cell culture).

### 2.3 Animals

Male wild-type (WT) C57BL/6-J JAX mice from Charles River (Leiden, Netherlands) were housed and handled according to the guidelines of the local ethics committee. Animal experiments were approved by the Animal Ethical Committee of the Radboud UMC Medical Center.

### 2.4 Induction of anti-GBM nephritis in mice, with/without administration of glycocalyx constituents or enoxaparin, and determination of kidney function

WT C57BL/6-J mice were injected i.v. in the tail vein with 8 mg rabbit anti-mouse GBM(36), alone or in combination with 50 µg of mGEnC glycocalyx, 50 µ g HS_glx_ or CS_glx_ fraction isolated from the mGEnC glycocalyx, and 50 µg enoxaparin or with sterile PBS. Mice were sacrificed after 2 h, 1, or 4 days, and each group comprised 4–5 mice. Urine was collected directly through bladder puncture or after 18 h metabolic cages. Harvested kidneys were fixed in 10% buffered formalin or snap-frozen in liquid nitrogen. Albumin concentration was measured by radial immunodiffusion (Mancini). Urinary creatinine and blood urea nitrogen (BUN) concentrations were determined enzymatically (Roche) in the Radboud UMC diagnostics facility.

### 2.5 Immunofluorescence staining

Frozen sections (2 µm) were fixed in ice-cold acetone for 10 min and stained essentially as described previously (Rops et al., 2007b). Directly labeled antibodies included goat anti-mouse C3c and fibrinogen-fluorescein isothiocyanate (FITC) (Nordic, Tilburg, Netherlands), goat anti-rabbit IgG Alexa-488 (Life Technologies, Breda, Netherlands), rat anti-mouse GR-1 (RB6.8C5)-FITC (BD Biosciences, Alphen aan de Rijn, Netherlands), and goat anti-Armenian hamster-Cy3 (Jackson ImmunoResearch Laboratories, West Grove, PA). Unlabeled primary antibodies included rat anti-mouse-CD68 (MCA 1957; Serotec, Oxford, United Kingdom) and hamster anti-agrin (MI91) (Raats et al., 1998). Sections were fixed with 1% paraformaldehyde–PBS and embedded in VectaShield mounting medium H-1000 (Brunschwig Chemie, Amsterdam, Netherlands). Goat anti-rabbit IgG, goat anti-mouse C3c, fibrinogen, and anti-HS scFv staining intensities were evaluated semi-qualitatively from 0 (no staining) to 10 (100% staining intensity inside the glomeruli) and averaged over 50 glomeruli. All quantitative observations were made by two independent observers on blinded sections. Glomerular influx of granulocytes was determined by counting the number of cells per 50 glomeruli.

### 2.6 Renal histology

Histological assessment of the kidneys was performed on 4-µm-thick paraffin sections that were stained using periodic acid–Schiff (PAS) reagent. Slide digitization was performed using a PANNORAMIC 1000 digital slide scanner (3DHistech, Budapest, Hungary) with a ×20 objective. The whole slide images (WSIs) were analyzed using CaseViewer 2.4 software (3DHistech, Budapest, Hungary). The histology of all glomeruli in a single kidney cross section (minimal 63 glomeruli) was evaluated in a blinded manner. The percentage of affected glomeruli, showing thrombosis and/or hyalinosis within the glomerular capillaries, was scored, and hereby, the percentage of the affected glomerular tuft area was measured.

### 2.7 Leukocyte binding assay

Confluent mGEnC/HRGECs in 96-well plates were stimulated as described previously. Primary human neutrophils were isolated from EDTA-whole blood by Ficoll density gradient centrifugation as described previously (Pieterse et al., 2016). Then, 6 x 10^5^ cells/mL (32Dcl3, primary neutrophils) were labeled with calcein-AM (25 μg/mL, Invitrogen) in PBS for 30 min at 37°C, washed in PBS, and resuspended in serum-free medium, as previously described (Rops et al., 2008). A total of 30,000–60,000 labeled cells were added to each well and incubated at 37°C for 30 min. Where indicated, cells were pre-incubated with 15–25 µg purified mGEnC HS_glx_ or 1 cm^2^ purified HRGEC HS_glx_ (3.125:1 ratio with the cultured cell layer) for 5 min. After binding, plates were filled with PBS and centrifuged twice upside down at ×300 g for 5 min. Cells were lysed with 100 µL of 50 mM Tris pH 8.3/0.1% SDS and transferred to a flat black-walled, clear-bottomed 96-well plate (Invitrogen), and fluorescence was measured (λ_ex_ 495 nm, λ_em_ 515 nm).

### 2.8 Recombinant protein binding in competition ELISAs

For protein binding to cells, confluent mGEnCs in 96-well plates were stimulated as described previously. Cells were washed with PBS and incubated for 1 h at 37°C and 5% CO_2_ with recombinant mouse (rm) protein: rmL-selectin (2 μg/mL, R&D Systems) and rmCD11b (10 μg/mL, R&D Systems), alone or protein pre-incubated with 15 µg mGEnC HS_glx_, or equivalent fractionated material F1 (16.7%, ∼2.5 µg) or F2 (28%, ∼4.25 µg) (Supplementary Figure S7) for 5 min. Cells were washed twice with PBS before protein binding was probed with antibodies: anti-mouse L-selectin-biotin (1:1,000, R&D Systems) and anti-mouse CD11b-biotin (1:4,000, eBioscience), on ice for 30 min, followed by streptavidin-HRP (1 μg/mL, Thermo Scientific) for further 30 min on ice. Plates were washed twice with PBS between each step. Cells were incubated with 100 µL of ×1 TMB substrate solution (Invitrogen), and the reaction was terminated with 100 µL of 1 N H_2_SO_4._ Plates were analyzed for absorbance at 450 nm using an ELISA reader (Bio-Rad Benchmark Plus). For cell-free direct protein binding, recombinant human (rh) protein: L-selectin/CD62L Fc Chimera (4 μg/mL, R&D Systems) and integrin alpha M beta 2 protein (CD11b) (3 μg/mL, R&D Systems) were immobilized overnight at room temperature onto Protein G (Thermo Scientific) and MaxiSorp NUNC-Immuno (Thermo Scientific), respectively. Plates were blocked with 2% BSA/10 μg/mL mouse IgG protein (Sigma) and 2% BSA, respectively, for 1 h at room temperature before incubation with 1 cm^2^ purified HRGEC HS_glx_. Plates were washed twice with PBS and probed for HS binding with antibodies diluted in 1% BSA: anti-HS ScFv HS4C3 (1:100) (van Kuppevelt et al., 1998), followed by anti-VSV-peroxidase (1:2,000, Sigma). Plates were analyzed using TMB and 1 N H_2_SO_4_, as described previously.

### 2.9 Capillary electrophoresis-mass spectrometry

Capillary electrophoresis-mass spectrometry and HS oligosaccharide structural prediction were performed as previously described (Sanderson et al., 2018).

### 2.10 Statistical analysis

Values are expressed as means ± S.E.M., and significance between two groups was evaluated by Student’s t-tests. Significance between more than two groups was evaluated by one-way ANOVA with Dunnett’s *post hoc* test using GraphPad Prism, version 8 software (GraphPad Software, Inc., San Diego, CA).

## 3 Results

### 3.1 Isolated glomerular endothelial glycocalyx or enoxaparin does not affect the induction of anti-GBM-induced glomerulonephritis in mice

Since we hypothesized that exogenous application of mGEnC-derived glycocalyx could have beneficial effects on the outcome of experimental glomerulonephritis, we first isolated total glycocalyx from cultured unstimulated mGEnC glycocalyx and subsequently separated HS and CS (termed HSglx and CSglx, respectively). All glycocalyx preparations, and the low-molecular-weight heparin, enoxaparin, as a proxy control, were tested for their efficacy in the anti-mouse GBM rabbit Ig-induced experimental glomerulonephritis model, which is primarily driven by the rapid (peaking at 2 h) glomerular influx of granulocytes (Assmann et al., 1985; Schrijver et al., 1990).

First, we evaluated whether the administration of mGEnC-derived total glycocalyx, HS_glx_, CS_glx_, or enoxaparin could affect the induction of the rabbit anti-mouse GBM glomerulonephritis model. Rabbit anti-mouse GBM IgG binding was comparable in all groups at every time point assessed (2 h, 1, and 4 days) for rabbit anti-mouse GBM IgG-injected mice (Supplementary Figure S1A). Similarly, complement activation was not affected by the administration of mGEnC-derived total glycocalyx, HS_glx_, CS_glx_, or enoxaparin (Supplementary Figure S1B). Thus, the induction of anti-GBM glomerulonephritis was not affected by any of the GAG preparations administered.

### 3.2 Administration of mGEnC-derived glycocalyx reduces glomerular fibrin deposition and albuminuria in experimental anti-GBM glomerulonephritis

**FIGURE 1:**
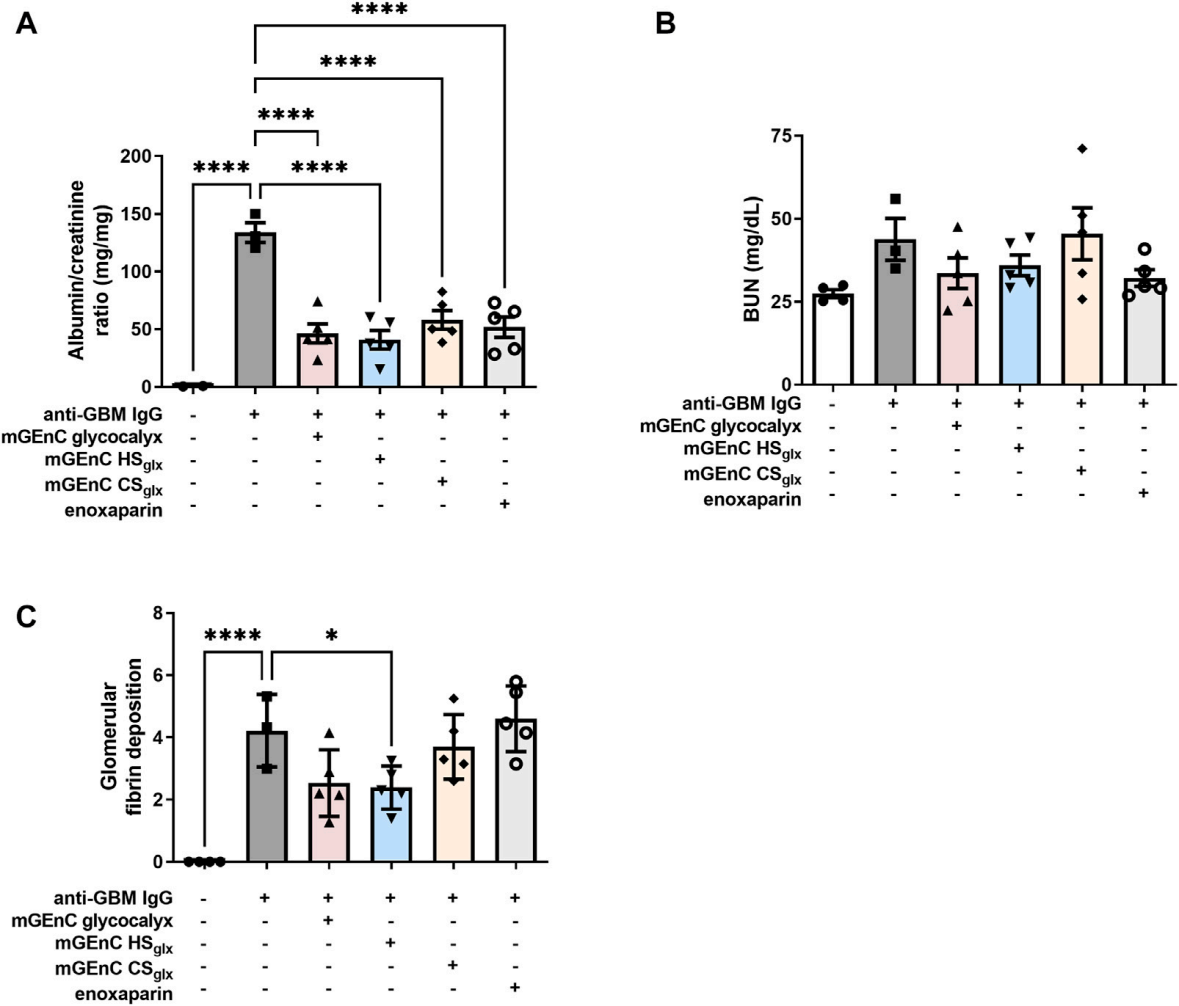
mGEnC-derived glycocalyx components and enoxaparin reduce albuminuria and glomerular fibrin deposition in anti-GBM-induced glomerulonephritis. **(A)** Albuminuria after 4 days of anti-GBM-induced glomerulonephritis in untreated mice and mice treated with 50 µg mGEnC glycocalyx, mGEnC HS_glx_, mGEnC CS_glx_, or enoxaparin. **(B)** Blood urea nitrogen (BUN) concentration after 4 days of anti-GBM nephritis. **(C)** Glomerular fibrin deposition, analyzed by immunofluorescence staining, after 4 days of anti-GBM nephritis. Fibrin deposition was scored semi-quantitatively between 0 and 10 based on the percentage of the glomerulus positive for fibrin. Per mouse, at least 25 glomeruli scored by two individual observers on blinded sections. Results are expressed as means ± S.E.M. from 3–5 mice per group in arbitrary units (a.u.). One-way ANOVA with Dunnett’s multiple comparison tests. *p ≤ 0.05 vs. anti-GBM IgG-injected mice, ****p < 0.0001 vs. anti-GBM IgG-injected mice.

Next, we measured albuminuria, blood urea nitrogen (BUN), as a measure for renal function, and glomerular fibrin deposition to assess whether administration of mGEnC glycocalyx, HS_glx_, CS_glx_, or enoxaparin influenced the outcome of the anti-GBM glomerulonephritis model. Albuminuria was significantly lower (∼3-fold) in mice treated with mGEnC total glycocalyx, HS_glx_, CS_glx_, or enoxaparin than control mice after 4 days of anti-GBM glomerulonephritis (Figure 1A). As expected, induction of anti-mouse GBM glomerulonephritis had not yet increased BUN values at indicated time points, since this only increases at day 8 (Rops et al., 2007b). Nevertheless, there seems to be a trend that GAG administration lowers BUN (Figure 1B). Furthermore, glomerular fibrin deposition was lower in mice treated with mGEnC total glycocalyx (*p* = 0.07) or HS_glx_ (*p* < 0.05), whereas CS_glx_ or enoxaparin had no effect (Figure 1C; Supplementary Figure S2). Notably, none of the administered GAG preparations influenced the kidney damage in our models, as measured by the percentage of affected glomeruli characterized mainly by thrombosis and hyalinosis within the glomerular capillaries (Supplementary Figure S3). Notably, we did not observe the formation of glomerular crescents or glomerulosclerosis, which is consistent with our model (Rops et al., 2007b). Hence, administration of mGEnC-derived total glycocalyx or mGEnC HS_glx_ is beneficial for renal outcome in anti-GBM-induced glomerulonephritis.

### 3.3 mGEnC-derived total glycocalyx and mGEnC HS_glx_ reduce glomerular granulocyte and macrophage influx in experimental anti-GBM glomerulonephritis

**FIGURE 2:**
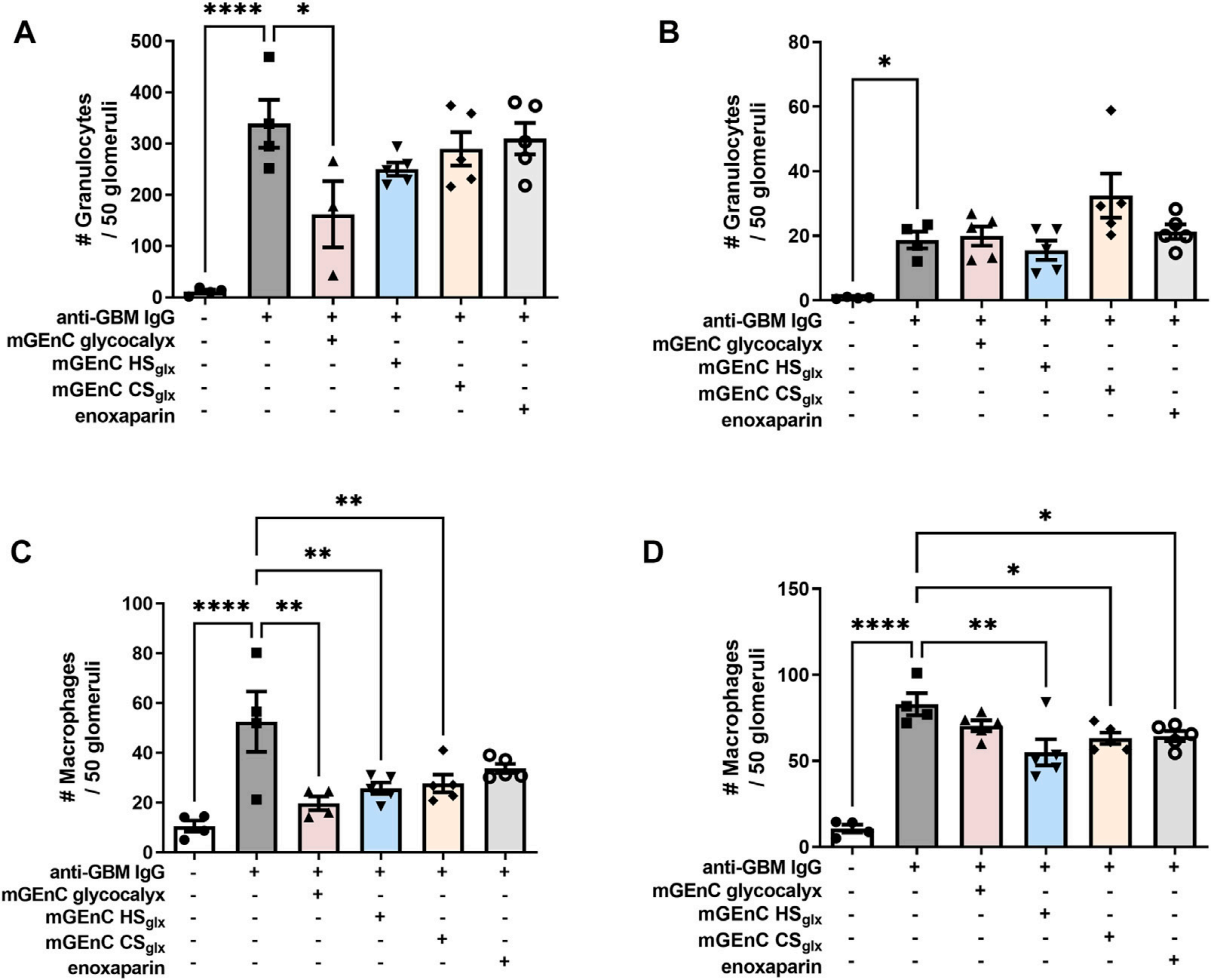
Administration of mGEnC glycocalyx and mGEnC HSglx reduce glomerular granulocyte and macrophage influx in anti-GBM glomerulonephritis. Glomerular granulocyte influx was analyzed by immunofluorescence staining at **(A)** 2 h and **(B)** 1 day after rabbit anti-GBM IgG administration in untreated mice and mice treated with 50 µg mGEnC glycocalyx, mGEnC HSglx, mGEnC CSglx, or enoxaparin. Glomerular macrophage influx, analyzed by immunofluorescence staining, **(C)** 2 h and **(D)** 1 day after rabbit anti-GBM IgG injection. Results are expressed as means ± S.E.M. from 4–5 mice per group. One-way ANOVA with Dunnett’s multiple comparison tests. *p ≤ 0.05, **p ≤ 0.01, ***p ≤ 0.001, ****p ≤ 0.0001 vs. anti-GBM-injected mice.

Our experimental anti-GBM-induced glomerulonephritis model is granulocyte-driven (Assmann et al., 1985; Schrijver et al., 1990) and characterized by the heterologous phase during which glomerular granulocyte influx peaks 2 h as a response to rabbit anti-mouse GBM IgG injection (Schrijver et al., 1990). The heterologous phase is followed by an autologous phase, starting approximately 4 days after induction of the model, during which self-antibodies against the injected rabbit IgG start to contribute to the disease progression. However, the level of initial glomerular PMN influx remains the key determinant for the severity of the disease with regard to renal outcome. Administration of mGEnC total glycocalyx or HS_glx_, respectively, reduced, or tended to reduce, glomerular granulocyte influx by approximately 52% ± 19% and 24% ± 6%, 2 h after anti-GBM IgG administration (Figure 2A; Supplementary Figure S4), while CS_glx_ (9% ± 13%) and enoxaparin CS_glx_ (8% ± 16%) did not affect glomerular granulocyte influx at 2 h (Figure 2A). After 1 day, glomerular granulocyte influx decreased more than 10-fold in all groups compared to the levels at 2 h, and there were no significant differences between the groups (Figure 2B; Supplementary Figure S4). In addition, we also evaluated glomerular macrophage influx after 2 h and 1 day (Figures 2C, D; Supplementary Figure S5). Similar to granulocytes, administration of mGEnC glycocalyx or mGEnC HS_glx_ reduced the glomerular influx of macrophages after 2 h by 62% ± 5% and 51% ± 4%, respectively, but CS_glx_ (47% ± 7%) and enoxaparin (36% ± 5%) also reduced macrophage presence, although this effect was not significant for enoxaparin (Figure 2C; Supplementary Figure S5). After 1 day, all treatments resulted in a lower glomerular presence of macrophages than the untreated anti-GBM glomerulonephritis group (Figure 2D), although this effect was not significant for mGEnC glycocalyx-treated mice. Therefore, administration of mGEnC-derived total glycocalyx or mGEnC HS_glx_ reduced glomerular influx of granulocytes and macrophages in anti-GBM-induced glomerulonephritis.

### 3.4 HS_glx_ reduces granulocyte binding to activated glomerular endothelial cells

**FIGURE 3:**
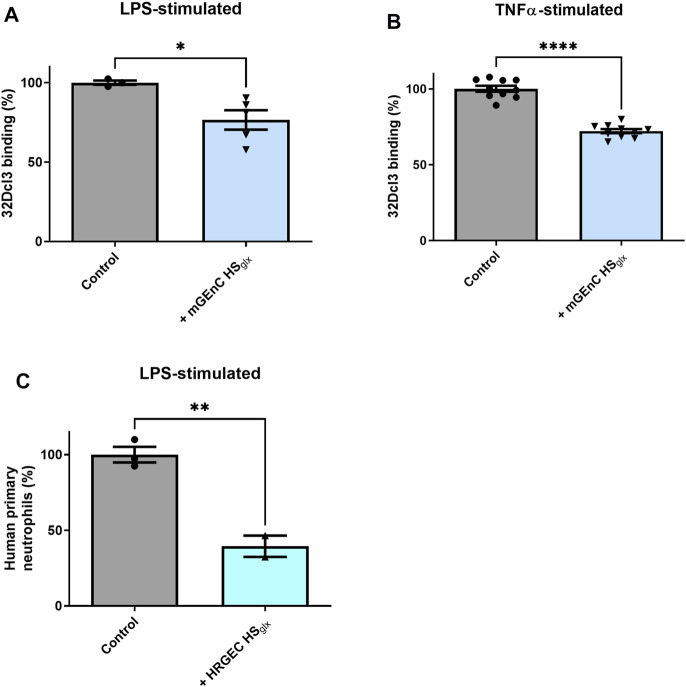
Purified glomerular endothelial cell-derived HS reduces granulocyte binding to activated mouse and human glomerular endothelial cells. PMN (32Dcl3) binding to **(A)** LPS- (N = 3–5) and **(B)** TNFα-stimulated mGEnCs (N = 10) in the absence or presence of 15–25 µg mGEnC HS_glx_. Unpaired *t*-test. * *p ≤* 0.05, **** *p ≤* 0.0001 vs. control. **(C)** Human primary neutrophil binding to LPS-stimulated primary human glomerular endothelial cells (HRGECs) in the absence or presence of 1 cm^2^ culture purified HRGEC HS_glx._ N = 2–3. Unpaired *t*-test. ** *p ≤* 0.01 vs. control.

In light of the inhibitory effect of HS_glx_ on glomerular influx of inflammatory cells in the anti-GBM glomerulonephritis model, we hypothesized that the granulocyte–glomerular endothelium interaction may have been inhibited by the administration of mGEnC HS_glx_. To investigate this, we performed binding studies of granulocytes to cultured TNFα- or LPS-activated mouse (mGEnC) or primary human (HRGEC) glomerular endothelial cells, using mGEnC HS_glx_ and HRGEC HS_glx_, respectively. Indeed, granulocyte binding was reduced in both culture models by application of purified HS_glx_ (Figures 3A–C). In short, the granulocyte–glomerular endothelium interaction is competitively inhibited by the addition of exogenous HS_glx_, and this mechanism seems to be conserved between mice and humans.

### 3.5 HS oligosaccharides derived from HS_glx_ inhibit L-selectin and CD11b binding to activated glomerular endothelial cells

**FIGURE 4:**
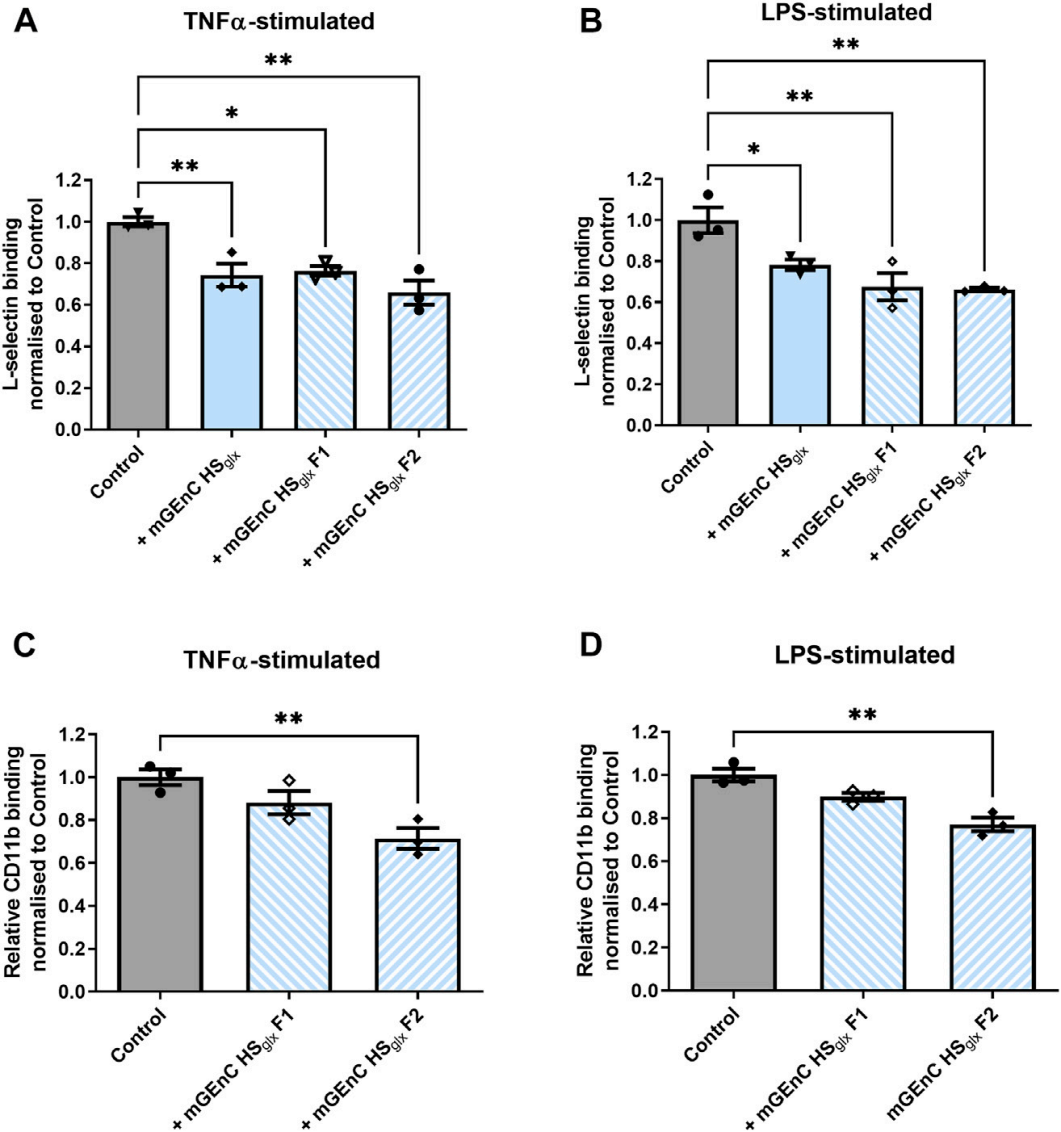
Binding of L-selectin and CD11b to activated glomerular endothelial cells is affected by specific mGEnCglx fractions. Recombinant mouse L-selectin binding to **(A)** TNFα- and **(B)** LPS-stimulated mGEnCs in the absence or presence of 15 µg of mGEnC HS_glx_ and size-exclusion fractions F1 (∼2.5 µg) or F2 (∼4.25 µg) from equivalent HS_glx_ starting material. Recombinant mouse CD11b binding to **(C)** TNFα- and **(D)** LPS-stimulated mGEnCs in the absence or presence of 15 µg mGEnC HS_glx_ and size-exclusion fractions F1 (∼2.5 µg) or F2 (∼4.25 µg) from equivalent HS_glx_ starting material. N = 3 for all experiments. One-way ANOVA with Dunnett’s multiple comparison tests. *p ≤ 0.05, **p ≤ 0.01 vs. control.

Both L-selectin and CD11b are expressed by granulocytes and macrophages and are well known to interact with HS (Diamond et al., 1995; Celie et al., 2005; Wang et al., 2005; Zen et al., 2009). We showed that purified HRGEC HS_glx_ binds to both recombinant human L-selectin and CD11b proteins *in vitro* (Supplementary Figure S6). Therefore, we investigated recombinant mouse L-selectin binding to activated mGEnC, either in the absence or presence of mGEnC HS_glx._ These experiments revealed that mGEnC HS_glx_ decreased L-selectin binding to activated mGEnC (Figures 4A, B). Next, we fractionated mGEnC HS_glx_ via size-exclusion chromatography (Supplementary Figure S7). To reduce the size of mGEnC F1 further, F1 was digested with heparinase III (Nader et al., 1999). It appears that both mGEnC-1 HS_glx_ F1 and mGEnC-1 HS_glx_ F2 inhibited L-selectin binding to activated mGEnC-1 (Figures 4A, B). Notably, mGEnC HS_glx_ F2 significantly inhibited adhesion of both binding of L-selectin and CD11b to activated mGEnCs, whereas mGEnC HS_glx_ F1 only inhibited L-selectin (Figures 4A–D).

To gather more information about HS oligosaccharide size and number of sulfates, we analyzed mGEnC HS_glx_ F2 by capillary electrophoresis-mass spectrometry (Sanderson et al., 2018) (Supplementary Figure S8 and Supplementary File 1). Six HS oligosaccharides were detected based on mass, ranging from tetra- to hexasaccharides with 2–7 sulfate groups (Table 1). In summary, short HS_glx_-derived oligosaccharides prevented binding of L-selectin or CD11b to glomerular endothelium, showing therapeutic potential for glomerulonephritis.

**TABLE 1:** Putative HS oligosaccharide structures in mGEnC HSglx fraction 2 (F2).^a^

Neutral mass	Number of				Theoretical mass	Mass error (ppm)	dp	Possible structure
	Uronic	Amino	N-Acetyl	SO_3_				
936.14125	2	2	2	2	936.1471	−6.28	4	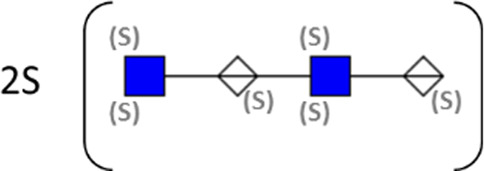 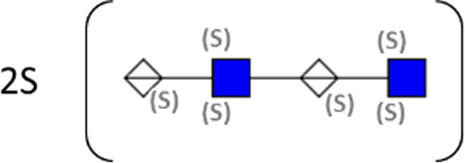
1,012.03405	2	2	0	4	1,012.04	−5.51	4	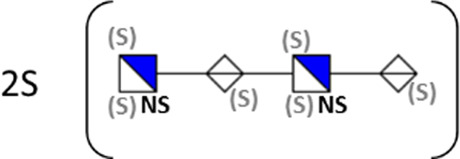 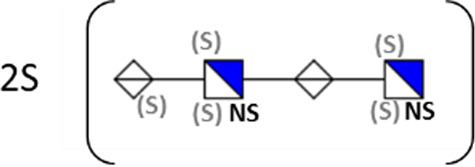
1,253.0585	2	3	0	5	1,253.065	−5.39	5	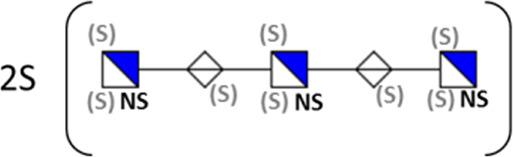
1,333.0149	2	3	0	6	1,333.022	−5.38	5	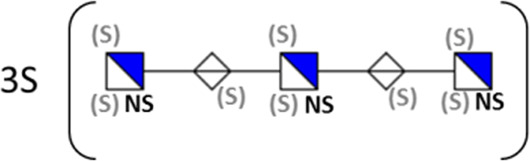
1,412.970675	2	3	0	7	1,412.979	−5.81	5	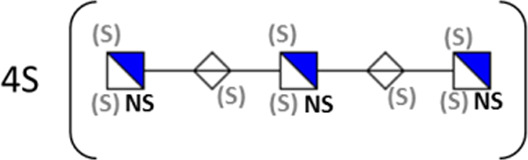
1,551.054675	3	3	1	6	1,551.065	−6.48	6	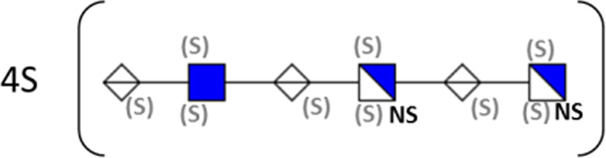 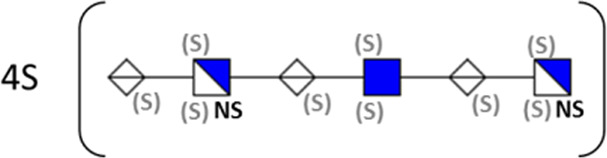 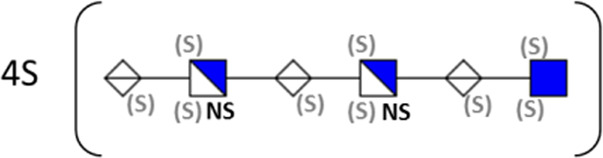 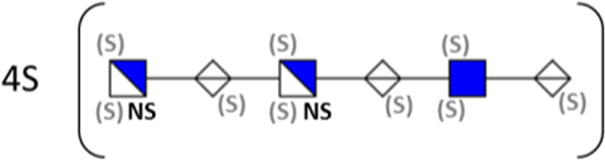 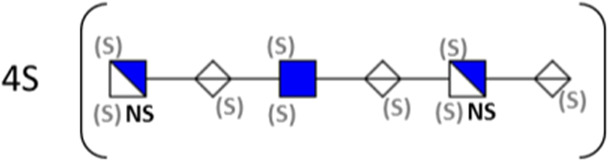 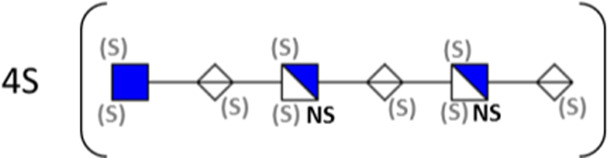

^a^ Mass spectrometry analysis of HS oligosaccharide species. Sulfated positionally assigned in black. The putative position of sulfate groups is in gray and in brackets. Number of unassigned sulfate groups in addition to assigned sulfate groups noted before the structure, i.e., 4S (pictorial structure). Uronic, uronic acid sugar; Amino, amino sugar, N-Acetyl, N-acetyl sugar; SO_3_, sulfate group; mass error, calculated mass balance error; ppm, parts per million; dp, degrees of polymerization; (S), potential sulfation site; NS, N-sulfate group.

## 4 Discussion

Glomerulonephritis may lead to chronic kidney disease, characterized by glomerular injury, proteinuria, and loss of kidney function. One of the major events during glomerulonephritis is the glomerular influx of leukocytes into the glomerulus, where leukocytes release effector molecules that cause tissue injury, leading to loss of kidney function. Previously, we and other research groups have described the importance of HS for the interaction of leukocytes with the glomerular endothelium (Diamond et al., 1995; Wang et al., 2002; Celie et al., 2005; Parish, 2005; Wang et al., 2005; Rops et al., 2008; Rops et al., 2014). Therefore, in this study, we hypothesized that the administration of purified glomerular endothelial glycocalyx, and in particular HS_glx,_ may interfere with the glomerular endothelium–leukocyte interaction, thereby providing therapeutic potential for patients with inflammatory kidney diseases. Our data showed that the administration of all GAG preparations reduced albuminuria, demonstrating that a GAG-based treatment can ameliorate glomerulonephritis *in vivo*.

Notably, the low-molecular-weight heparin, enoxaparin, which we used as a proxy control, was less protective than mGEnC HS_glx_ during anti-GBM-induced glomerulonephritis, despite the fact that enoxaparin has been shown to reduce leukocyte adhesion and chemokine binding to glomerular endothelium *in vitro* (Rops et al., 2008; van Gemst et al., 2018), and others have shown the protective effect of enoxaparin in lupus nephritis (Hedberg et al., 2013). However, none of these studies evaluated glomerular endothelial glycocalyx or HS_glx_, which, in our opinion, may have been even more effective than enoxaparin as shown in the current study. Our results strongly suggest that sequences within glomerular endothelial HS_glx_ are optimally suited for the therapeutic treatment of inflammatory glomerular diseases. HS and its domains provide a more heterogeneous source of structures than enoxaparin and other heparinoids (Esko and Selleck, 2002; Wang et al., 2002; Rops et al., 2007c). Additionally, HS contains the natural complementary motifs for HS–ligand interactions, as we observe *in vivo* (Koenig et al., 1998), and we hypothesized that the purified HS_glx_ competes for ligands involved in key steps for leukocyte extravasation, like L-selectin and CD11b. The specific HS–protein interaction is largely determined by HS fine structure, namely, the sulfation pattern in a given HS chain (Esko and Selleck, 2002). We have demonstrated previously that mGEnC HS_glx_ contains multiple domain structures (Rops et al., 2007c), thereby potentiating that mGEnC HS_glx_ could contain a range of HS oligosaccharide structures for interaction with multiple ligands. In this study, we focused on two well-known endothelial HS ligands, L-selectin and CD11b, both playing pivotal roles in the leukocyte adhesion cascade. Purified mGEnC HS_glx_ significantly inhibited both L-selectin and CD11b binding to TNFα- and LPS-stimulated mGEnCs, thereby suggesting that disruption of more than one HS–ligand interaction may have contributed to the therapeutic effects of total HS_glx_ observed *in vivo*. In addition to leukocyte–endothelium interaction, CD11b also plays roles in granulocyte phagocytosis, superoxide release, degranulation, and apoptosis, which may additionally be hampered upon HS_glx_ binding, thereby further contributing to the beneficial outcome of HS_glx_ injection observed *in vivo*. Notably, our *in vitro* experiments reveal that the mechanism of HS_glx_-mediated inhibition is conserved between mice and humans. Markedly, we identified one specific fraction of mGEnC HS_glx_ that prevented the binding of both L-selectin and CD11b *in vitro*. Mass spectrometry analysis of this fraction revealed six HS species (based on mass) that must contain the sequence motifs for binding to L-selectin and CD11b. Although we were able to partially decode these sequences, the number of possible sequences remains in the hundreds. Notably, deciphering the full sequence of an HS chain is an area of avid interest in the field of glycobiology but is a challenging task, not only largely due to the inherent heterogeneity of HS, but also because of the lack of chemically synthesized standards and isomeric separation methods for HS mass spectrometry analysis.

GAG-based therapeutics are a newly emerging class of drugs for the treatment of a wide range of diseases (Lindahl and Kjellen, 2013; Maciej-Hulme et al., 2018; Muralidar et al., 2021). The GAGs, heparin, HS, and their derivatives are one of the oldest and most widely used class of drugs in medicine owing to their valuable anti-coagulant properties. Recent advances in separation, detection, and mass spectrometry methods have facilitated reinvigoration in the development of HS-based drugs (van Gemst et al., 2016; Sanderson et al., 2018; Maciej-Hulme et al., 2023; Jain et al., 2021). In our proof-of-concept study, by systematic purification, fractionation, and identification, we considerably reduced the heterogeneity of glomerular glycocalyx HS species to only six species (based on mass) in a biologically active preparation. Moreover, the HS chains we identified were between tetra- and hexasaccharides in length, which are similar sizes to the FDA-approved pentasaccharide, fondaparinux (Petitou et al., 1997), thereby demonstrating that our approach yields bioactive oligosaccharides of synthesizable size. Notably, fondaparinux is the only heparin-derived sequence that has been linked to a single activity, i.e., antithrombin III binding, and is clinically applied as an anticoagulant. Together, our data demonstrate the potential of HS-based drugs to treat glomerulonephritis. The next step in the drug development pipeline is to structurally identify bioactive compounds in our active preparation, followed by chemoenzymatic synthesis of corresponding sequences.

In conclusion, HS_glx_ was identified as the GAG preparation from glomerular endothelial cells with the most promising therapeutic activity to attenuate experimental glomerulonephritis, in which HS oligosaccharides contained significant inhibitory activity for leukocyte binding to endothelium. Our data strongly support the application of HS-based therapeutics inspired by native glomerular endothelial HS_glx_ for glomerulonephritis and justify their further development for patients with (acute) inflammatory glomerular diseases.

## Data Availability

The original contributions presented in the study are included in the article/Supplementary Material; further inquiries can be directed to the corresponding author.

## References

[B1] Assmann K. J. Tangelder M. M. Lange W. P. Schrijver G. Koene R. A. (1985). Anti-GBM nephritis in the mouse: Severe proteinuria in the heterologous phase. Virchows Archiv A, Pathological Anat. Histopathol. 406 (3), 285–299. 10.1007/BF00704298 3923705

[B2] Bennett K. L. Jackson D. G. Simon J. C. Tanczos E. Peach R. Modrell B. (1995). CD44 isoforms containing exon V3 are responsible for the presentation of heparin-binding growth factor. J. Cell. Biol. 128 (4), 687–698. 10.1083/jcb.128.4.687 7532176 PMC2199889

[B3] Butcher E. C. (1991). Leukocyte-endothelial cell recognition: Three (or more) steps to specificity and diversity. Cell. 67 (6), 1033–1036. 10.1016/0092-8674(91)90279-8 1760836

[B4] Celie J. W. Keuning E. D. Beelen R. H. Drager A. M. Zweegman S. Kessler F. L. (2005). Identification of L-selectin binding heparan sulfates attached to collagen type XVIII. J. Biol. Chem. 280 (29), 26965–26973. 10.1074/jbc.M502188200 15917223

[B5] Dennissen M. A. Jenniskens G. J. Pieffers M. Versteeg E. M. Petitou M. Veerkamp J. H. (2002). Large, tissue-regulated domain diversity of heparan sulfates demonstrated by phage display antibodies. J. Biol. Chem. 277 (13), 10982–10986. 10.1074/jbc.M104852200 11790764

[B6] Diamond M. S. Alon R. Parkos C. A. Quinn M. T. Springer T. A. (1995). Heparin is an adhesive ligand for the leukocyte integrin Mac-1 (CD11b/CD1). J. Cell. Biol. 130 (6), 1473–1482. 10.1083/jcb.130.6.1473 7559767 PMC2120570

[B7] Elhadj S. Mousa S. A. Forsten-Williams K. (2002). Chronic pulsatile shear stress impacts synthesis of proteoglycans by endothelial cells: Effect on platelet aggregation and coagulation. J. Cell. Biochem. 86 (2), 239–250. 10.1002/jcb.10226 12111993

[B8] Esko J. D. Selleck S. B. (2002). Order out of chaos: Assembly of ligand binding sites in heparan sulfate. Annu. Rev. Biochem. 71, 435–471. 10.1146/annurev.biochem.71.110601.135458 12045103

[B9] Gao L. Lipowsky H. H. (2010). Composition of the endothelial glycocalyx and its relation to its thickness and diffusion of small solutes. Microvasc. Res. 80 (3), 394–401. 10.1016/j.mvr.2010.06.005 20600162 PMC2962421

[B10] Guimond S. E. Puvirajesinghe T. M. Skidmore M. A. Kalus I. Dierks T. Yates E. A. (2009). Rapid purification and high sensitivity analysis of heparan sulfate from cells and tissues TOWARD GLYCOMICS PROFILING. J. Biol. Chem. 284 (38), 25714–25722. 10.1074/jbc.M109.032755 19596853 PMC2757973

[B11] Handel T. M. Johnson Z. Crown S. E. Lau E. K. Proudfoot A. E. (2005). Regulation of protein function by glycosaminoglycans--as exemplified by chemokines. Annu. Rev. Biochem. 74, 385–410. 10.1146/annurev.biochem.72.121801.161747 15952892

[B12] Hedberg A. Kanapathippillai P. Rekvig O. P. Fenton K. A. (2013). LMW heparin prevents increased kidney expression of proinflammatory mediators in (NZBxNZW)F1 mice. Clin. Dev. Immunol. 2013, 791262. 10.1155/2013/791262 24151519 PMC3789300

[B13] Iozzo R. V. (2001). Heparan sulfate proteoglycans: Intricate molecules with intriguing functions. J. Clin. investigation 108 (2), 165–167. 10.1172/JCI13560 PMC203034 11457866

[B14] Iozzo R. V. (2005). Basement membrane proteoglycans: From cellar to ceiling. Nat. Rev. Mol. Cell. Biol. 6 (8), 646–656. 10.1038/nrm1702 16064139

[B15] Jain P. Shanthamurthy C. D. Leviatan Ben-Arye S. Woods R. J. Kikkeri R. Padler-Karavani V. (2021). Discovery of rare sulfated N-unsubstituted glucosamine based heparan sulfate analogs selectively activating chemokines. Chem. Sci. 12 (10), 3674–3681. 10.1039/d0sc05862a 33889380 PMC8025211

[B16] Jenniskens G. J. Oosterhof A. Brandwijk R. Veerkamp J. H. van Kuppevelt T. H. (2000). Heparan sulfate heterogeneity in skeletal muscle basal lamina: Demonstration by phage display-derived antibodies. J. Neurosci. official J. Soc. Neurosci. 20 (11), 4099–4111. 10.1523/JNEUROSCI.20-11-04099.2000 PMC6772625 10818145

[B17] Koenig A. Norgard-Sumnicht K. Linhardt R. Varki A. (1998). Differential interactions of heparin and heparan sulfate glycosaminoglycans with the selectins. Implications for the use of unfractionated and low molecular weight heparins as therapeutic agents. J. Clin. investigation 101 (4), 877–889. 10.1172/JCI1509 PMC508636 9466983

[B18] Lindahl U. Kjellen L. (2013). Pathophysiology of heparan sulphate: Many diseases, few drugs. J. Intern Med. 273 (6), 555–571. 10.1111/joim.12061 23432337

[B19] Maciej-Hulme M. L. Skidmore M. A. Price H. P. (2018). The role of heparan sulfate in host macrophage infection by Leishmania species. Biochem. Soc. Trans. 46 (4), 789–796. 10.1042/BST20170398 29934302

[B20] Maciej-Hulme M. L. Leprince A. Lavin A. Guimond S. Turnbull J. Pelletier J. (2023). High sensitivity (zeptomole) detection of BODIPY heparan sulfate (HS) disaccharides by ion-paired RP-HPLC and LIF detection enables analysis of HS from mosquito midguts. Anal. Methods. 15 (11), 1461–1469. 10.1101/20200121913954 36876452 PMC10019443

[B21] Marr H. S. Basalamah M. A. Edgell C. J. (1997). Endothelial cell expression of testican mRNA. Endothelium J. endothelial Cell. Res. 5 (3), 209–219. 10.3109/10623329709053399 9272383

[B22] Morimoto-Tomita M. Uchimura K. Werb Z. Hemmerich S. Rosen S. D. (2002). Cloning and characterization of two extracellular heparin-degrading endosulfatases in mice and humans. J. Biol. Chem. 277 (51), 49175–49185. 10.1074/jbc.M205131200 12368295 PMC2779716

[B23] Muralidar S. Gopal G. Visaga Ambi S. (2021). Targeting the viral-entry facilitators of SARS-CoV-2 as a therapeutic strategy in COVID-19. J. Med. Virol. 93, 5260–5276. 10.1002/jmv.27019 33851732 PMC8251167

[B24] Nader H. B. Kobayashi E. Y. Chavante S. F. Tersariol I. L. Castro R. A. Shinjo S. K. (1999). New insights on the specificity of heparin and heparan sulfate lyases from Flavobacterium heparinum revealed by the use of synthetic derivatives of K5 polysaccharide from *E. coli* and 2-O-desulfated heparin. Glycoconj J. 16 (6), 265–270. 10.1023/a:1007057826179 10579695

[B25] Parish C. R. (2005). Heparan sulfate and inflammation. Nat. Immunol. 6 (9), 861–862. 10.1038/ni0905-861 16116461

[B26] Parish C. R. (2006). The role of heparan sulphate in inflammation. Nat. Rev. Immunol. 6 (9), 633–643. 10.1038/nri1918 16917509

[B27] Petitou M. Duchaussoy P. Jaurand G. Gourvenec F. Lederman I. Strassel J. M. (1997). Synthesis and pharmacological properties of a close analogue of an antithrombotic pentasaccharide (SR 90107A/ORG 31540). J. Med. Chem. 40 (11), 1600–1607. 10.1021/jm960726z 9171870

[B28] Pieterse E. Rother N. Yanginlar C. Hilbrands L. B. van der Vlag J. (2016). Neutrophils discriminate between lipopolysaccharides of different bacterial sources and selectively release neutrophil extracellular traps. Front. Immunol. 7, 484. 10.3389/fimmu.2016.00484 27867387 PMC5095130

[B29] Raats C. J. Bakker M. A. Hoch W. Tamboer W. P. Groffen A. J. van den Heuvel L. P. (1998). Differential expression of agrin in renal basement membranes as revealed by domain-specific antibodies. J. Biol. Chem. 273 (28), 17832–17838. 10.1074/jbc.273.28.17832 9651386

[B30] Reitsma S. Slaaf D. W. Vink H. van Zandvoort M. A. oude Egbrink M. G. (2007). The endothelial glycocalyx: Composition, functions, and visualization. Pflugers Archiv Eur. J. physiology 454 (3), 345–359. 10.1007/s00424-007-0212-8 17256154 PMC1915585

[B31] Rops A. L. van der Vlag J. Lensen J. F. Wijnhoven T. J. van den Heuvel L. P. van Kuppevelt T. H. (2004). Heparan sulfate proteoglycans in glomerular inflammation. Kidney Int. 65 (3), 768–785. 10.1111/j.1523-1755.2004.00451.x 14871397

[B32] Rops A. L. van der Vlag J. Jacobs C. W. Dijkman H. B. Lensen J. F. Wijnhoven T. J. (2004). Isolation and characterization of conditionally immortalized mouse glomerular endothelial cell lines. Kidney Int. 66 (6), 2193–2201. 10.1111/j.1523-1755.2004.66009.x 15569308

[B33] Rops A. L. Jacobs C. W. Linssen P. C. Boezeman J. B. Lensen J. F. Wijnhoven T. J. (2007). Heparan sulfate on activated glomerular endothelial cells and exogenous heparinoids influence the rolling and adhesion of leucocytes. Nephrol. Dial. Transplant. 22 (4), 1070–1077. official publication of the European Dialysis and Transplant Association - European Renal Association. 10.1093/ndt/gfl801 17255131

[B34] Rops A. L. Gotte M. Baselmans M. H. van den Hoven M. J. Steenbergen E. J. Lensen J. F. (2007). Syndecan-1 deficiency aggravates anti-glomerular basement membrane nephritis. Kidney Int. 72 (10), 1204–1215. 10.1038/sj.ki.5002514 17805240

[B35] Rops A. L. van den Hoven M. J. Bakker M. A. Lensen J. F. Wijnhoven T. J. van den Heuvel L. P. (2007). Expression of glomerular heparan sulphate domains in murine and human lupus nephritis. Nephrol. Dial. Transplant. 22 (7), 1891–1902. official publication of the European Dialysis and Transplant Association - European Renal Association. 10.1093/ndt/gfm194 17550924

[B36] Rops A. L. van den Hoven M. J. Baselmans M. M. Lensen J. F. Wijnhoven T. J. van den Heuvel L. P. (2008). Heparan sulfate domains on cultured activated glomerular endothelial cells mediate leukocyte trafficking. Kidney Int. 73 (1), 52–62. 10.1038/sj.ki.5002573 17914352

[B37] Rops A. L. W. M. M. Loeven M. A. van Gemst J. J. Eversen I. Van Wijk X. M. Dijkman H. B. (2014). Modulation of heparan sulfate in the glomerular endothelial glycocalyx decreases leukocyte influx during experimental glomerulonephritis. Kidney Int. 86, 932–942. 10.1038/ki.2014.115 24759151

[B38] Sanderson P. Stickney M. Leach F. E. 3rd Xia Q. Yu Y. Zhang F. (2018). Heparin/heparan sulfate analysis by covalently modified reverse polarity capillary zone electrophoresis-mass spectrometry. J. Chromatogr. A 1545, 75–83. 10.1016/j.chroma.2018.02.052 29501428 PMC5862776

[B39] Saphire A. C. Bobardt M. D. Zhang Z. David G. Gallay P. A. (2001). Syndecans serve as attachment receptors for human immunodeficiency virus type 1 on macrophages. J. virology 75 (19), 9187–9200. 10.1128/JVI.75.19.9187-9200.2001 11533182 PMC114487

[B40] Schlondorff D. Nelson P. J. Luckow B. Banas B. (1997). Chemokines and renal disease. Kidney Int. 51 (3), 610–621. 10.1038/ki.1997.90 9067891

[B41] Schrijver G. Bogman M. J. Assmann K. J. de Waal R. M. Robben H. C. van Gasteren H. (1990). Anti-GBM nephritis in the mouse: Role of granulocytes in the heterologous phase. Kidney Int. 38 (1), 86–95. 10.1038/ki.1990.171 2385089

[B42] Taylor K. R. Gallo R. L. (2006). Glycosaminoglycans and their proteoglycans: Host-associated molecular patterns for initiation and modulation of inflammation. FASEB J. official Publ. Fed. Am. Soc. Exp. Biol. 20 (1), 9–22. 10.1096/fj.05-4682rev 16394262

[B43] van de Lest C. H. Versteeg E. M. Veerkamp J. H. van Kuppevelt T. H. (1994). Quantification and characterization of glycosaminoglycans at the nanogram level by a combined azure A-silver staining in agarose gels. Anal. Biochem. 221 (2), 356–361. 10.1006/abio.1994.1425 7529008

[B44] van Gemst J. J. Loeven M. A. de Graaf M. J. Berden J. H. Rabelink T. J. Smit C. H. (2016). RNA contaminates glycosaminoglycans extracted from cells and tissues. PLoS One 11 (11), e0167336. 10.1371/journal.pone.0167336 27898729 PMC5127559

[B45] van Gemst J. J. Kouwenberg M. Rops A. van Kuppevelt T. H. Berden J. H. Rabelink T. J. (2018). Differential binding of chemokines CXCL1, CXCL2 and CCL2 to mouse glomerular endothelial cells reveals specificity for distinct heparan sulfate domains. PLoS One 13 (9), e0201560. 10.1371/journal.pone.0201560 30248108 PMC6152867

[B46] van Kuppevelt T. H. Dennissen M. A. van Venrooij W. J. Hoet R. M. Veerkamp J. H. (1998). Generation and application of type-specific anti-heparan sulfate antibodies using phage display technology. Further evidence for heparan sulfate heterogeneity in the kidney. J. Biol. Chem. 273 (21), 12960–12966. 10.1074/jbc.273.21.12960 9582329

[B47] Wang L. Brown J. R. Varki A. Esko J. D. (2002). Heparin's anti-inflammatory effects require glucosamine 6-O-sulfation and are mediated by blockade of L- and P-selectins. J. Clin. investigation 110 (1), 127–136. 10.1172/JCI14996 PMC151027 12093896

[B48] Wang L. Fuster M. Sriramarao P. Esko J. D. (2005). Endothelial heparan sulfate deficiency impairs L-selectin- and chemokine-mediated neutrophil trafficking during inflammatory responses. Nat. Immunol. 6 (9), 902–910. 10.1038/ni1233 16056228

[B49] Wong S. H. Hamel L. Chevalier S. Philip A. (2000). Endoglin expression on human microvascular endothelial cells association with betaglycan and formation of higher order complexes with TGF-beta signalling receptors. Eur. J. Biochem./FEBS 267 (17), 5550–5560. 10.1046/j.1432-1327.2000.01621.x 10951214

[B50] Xia G. Chen J. Tiwari V. Ju W. Li J. P. Malmstrom A. (2002). Heparan sulfate 3-O-sulfotransferase isoform 5 generates both an antithrombin-binding site and an entry receptor for herpes simplex virus, type 1. J. Biol. Chem. 277 (40), 37912–37919. 10.1074/jbc.M204209200 12138164

[B51] Zen K. Liu D. Q. Li L. M. Chen C. X. Guo Y. L. Ha B. (2009). The heparan sulfate proteoglycan form of epithelial CD44v3 serves as a CD11b/CD18 counter-receptor during polymorphonuclear leukocyte transepithelial migration. J. Biol. Chem. 284 (6), 3768–3776. 10.1074/jbc.M807805200 19073595 PMC2635047

