# Supplementary figures and images for: Glomerular endothelial glycocalyx-derived heparan sulfate inhibits glomerular leukocyte influx and attenuates experimental glomerulonephritis
Source: Front Mol Biosci. 2023 Jun 1;10:1177560. doi: 10.3389/fmolb.2023.1177560 (PMC10267401; doi:10.3389/fmolb.2023.1177560)

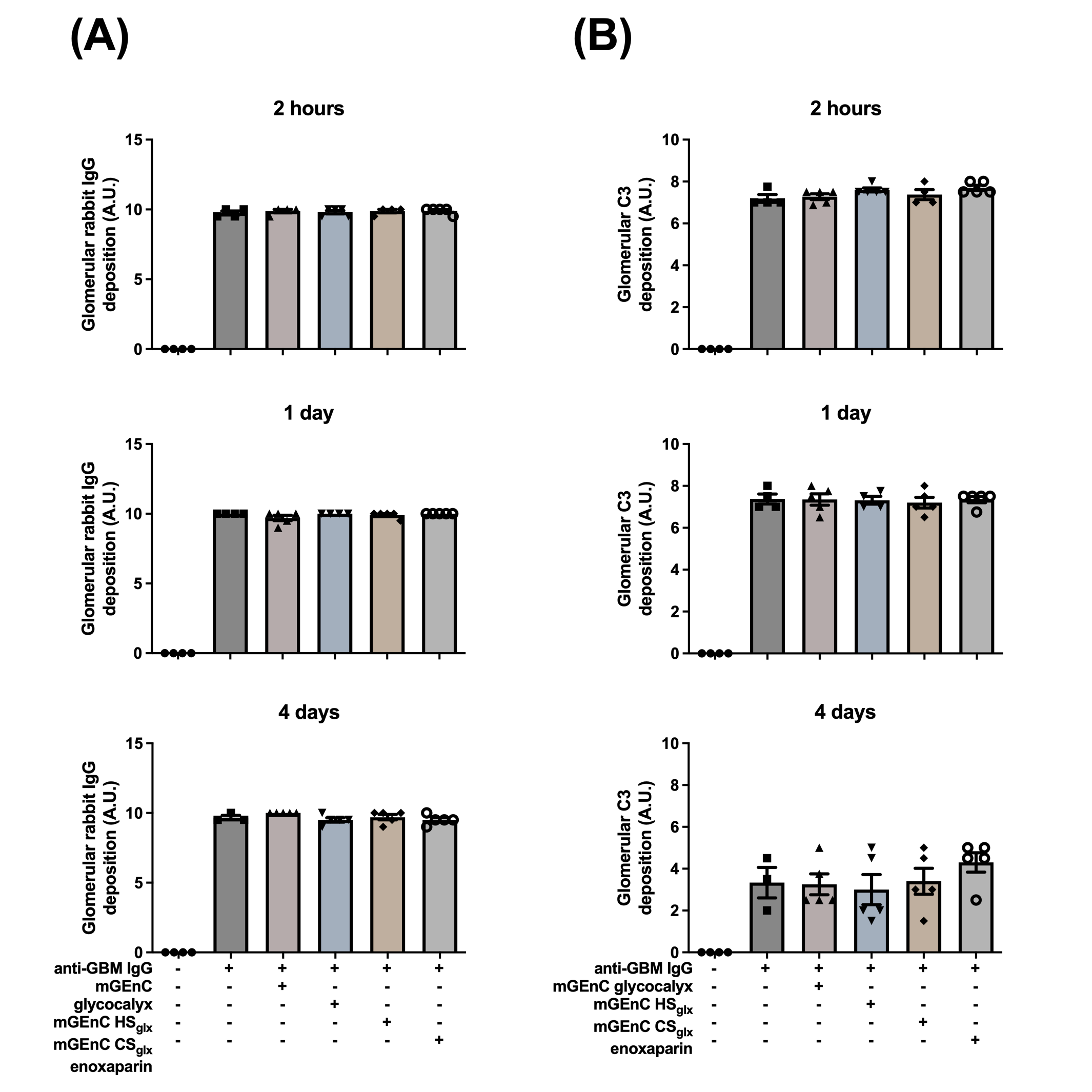

Supplement: Supplementary file 2 [file Image1.TIFF]

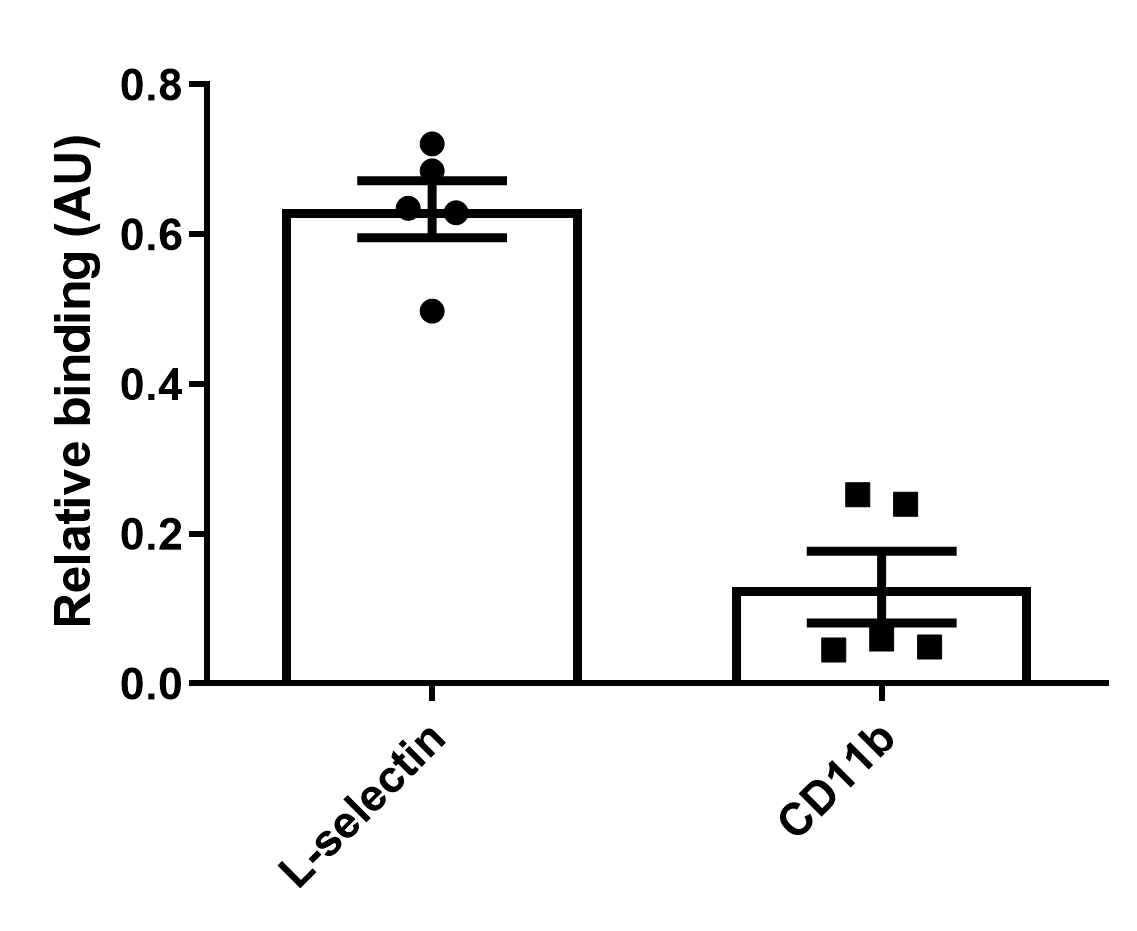

Supplement: Supplementary file 3 [file Image6.TIF]

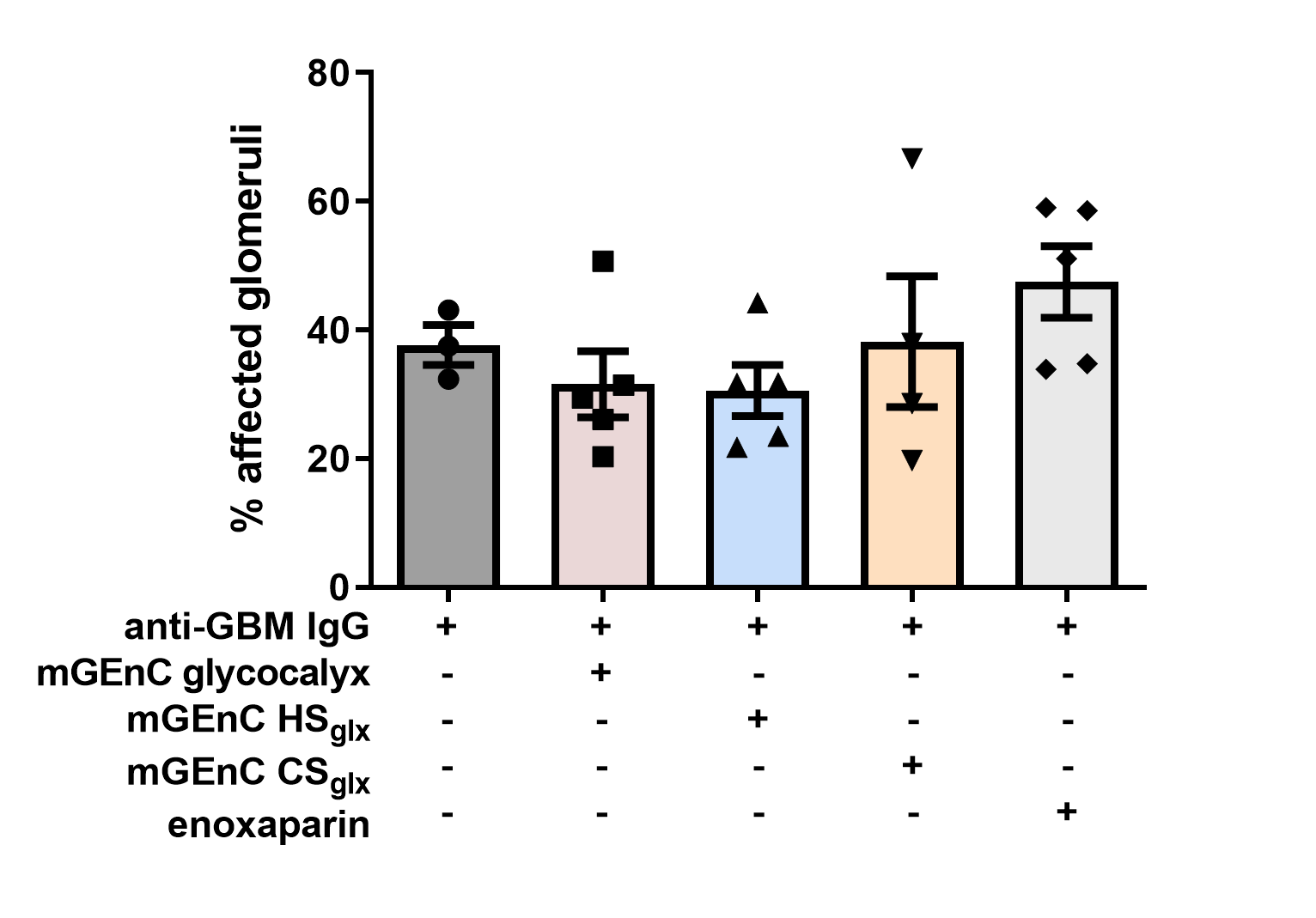

Supplement: Supplementary file 5 [file Image3.TIF]

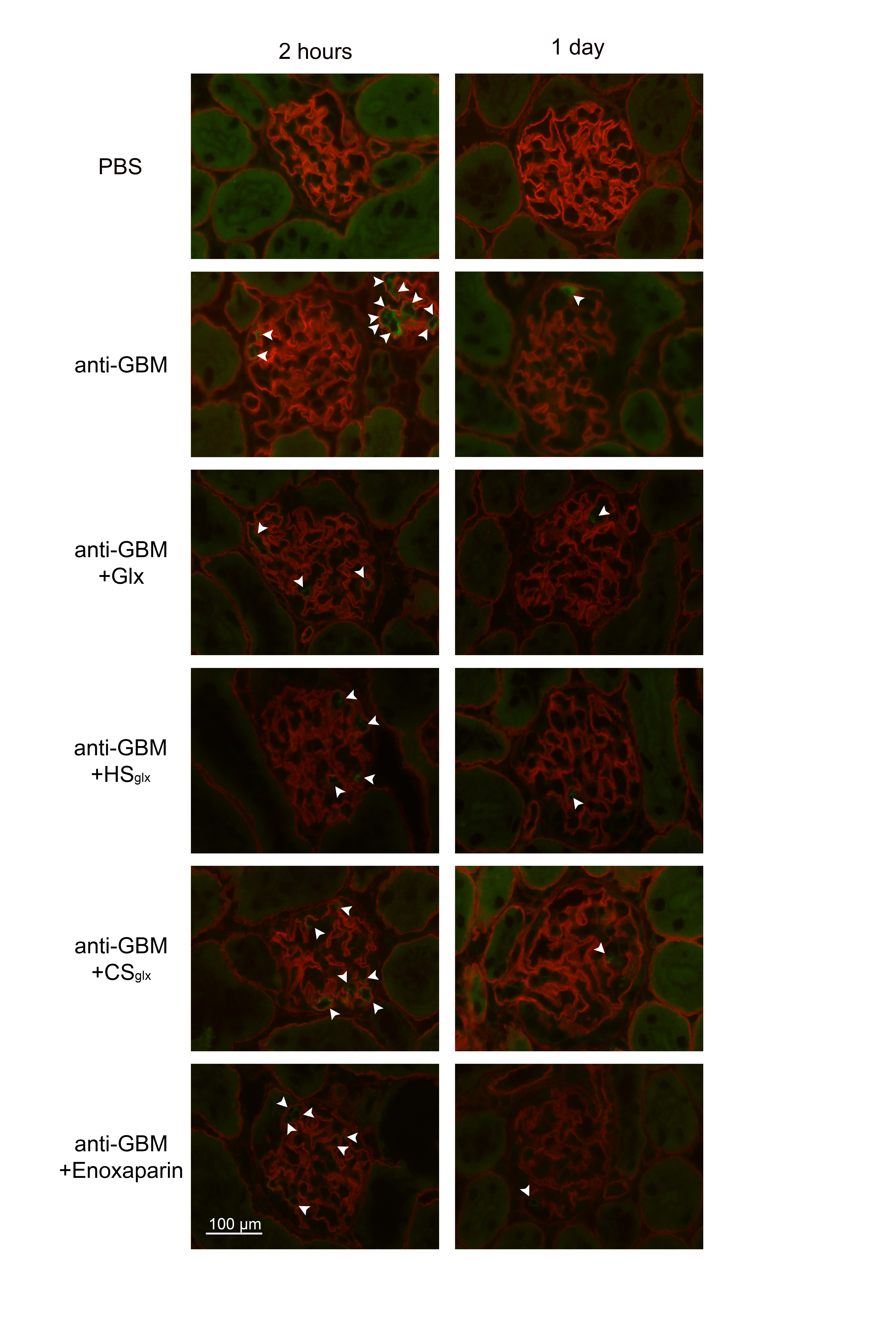

Supplement: Supplementary file 6 [file Image4.TIF]

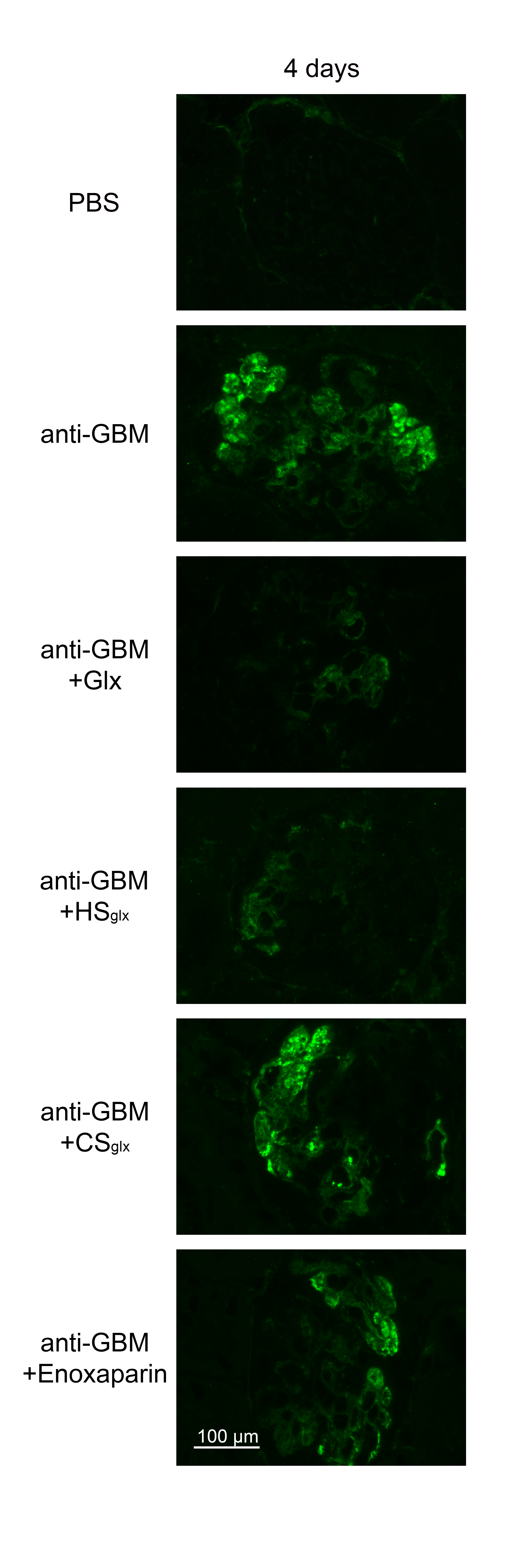

Supplement: Supplementary file 7 [file Image2.TIF]

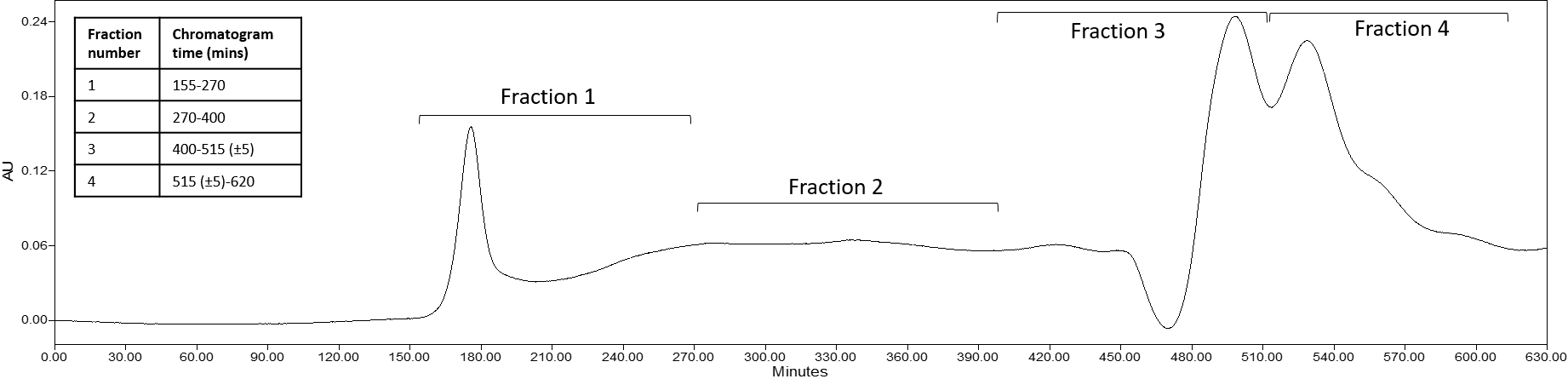

Supplement: Supplementary file 8 [file Image7.TIF]

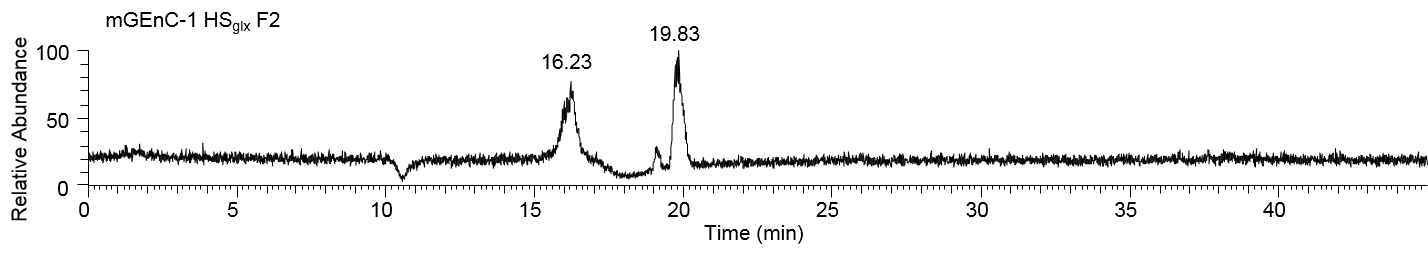

Supplement: Supplementary file 9 [file Image8.TIF]

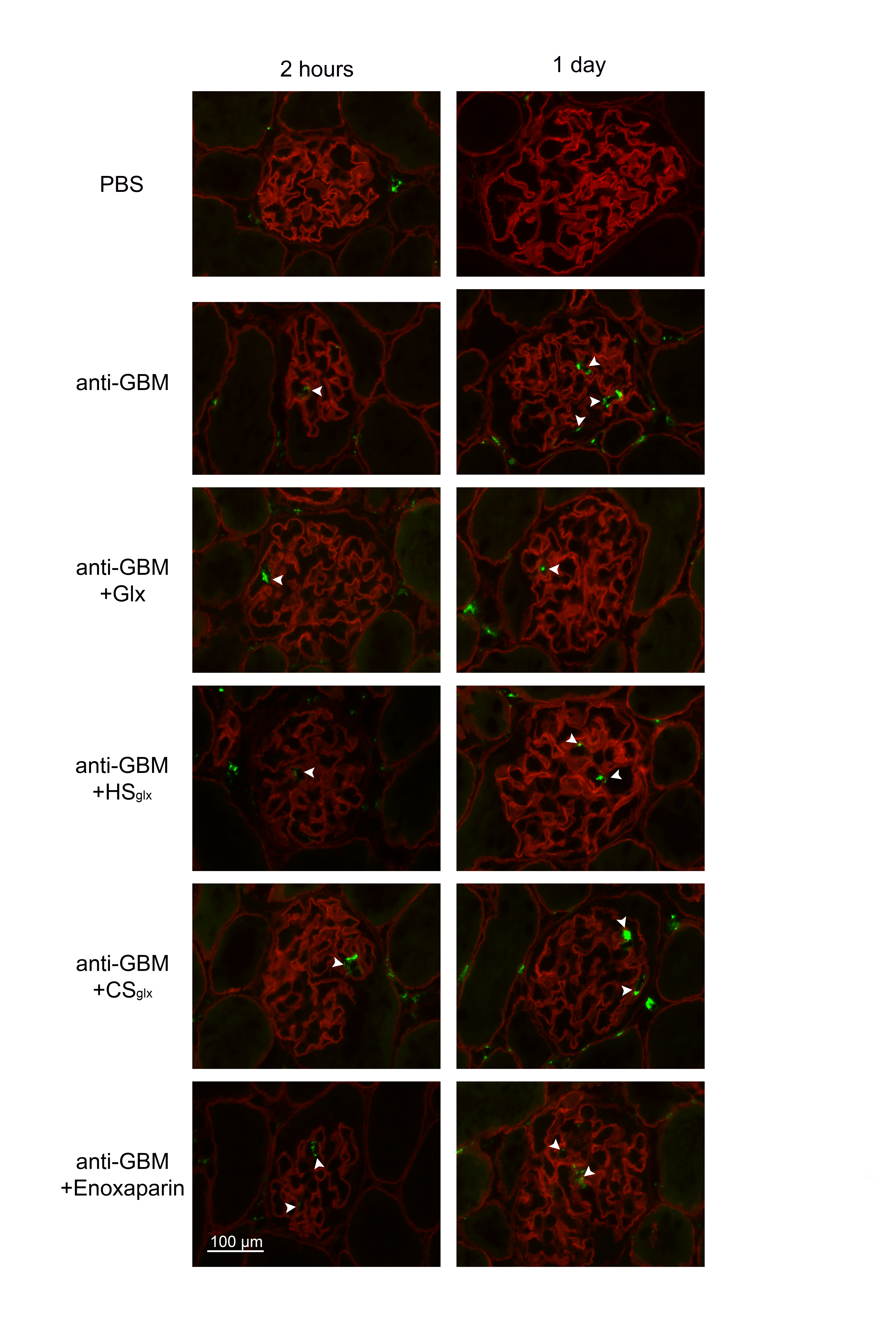

Supplement: Supplementary file 10 [file Image5.TIF]
