# Supplementary material for: Glomerular endothelial glycocalyx-derived heparan sulfate inhibits glomerular leukocyte influx and attenuates experimental glomerulonephritis
Source: Front Mol Biosci. 2023 Jun 1;10:1177560. doi: 10.3389/fmolb.2023.1177560 (PMC10267401; doi:10.3389/fmolb.2023.1177560)
Supplement: Supplementary file 4 [file Table1.DOCX]

Supplementary Material

Glomerular endothelial glycocalyx-derived heparan sulfate inhibits glomerular leukocyte influx and attenuates experimental glomerulonephritis

Marissa. L. Maciej-Hulme^1^*, Jasper J. van Gemst^1^*, Patience Sanderson^2^, Angelique L.W.M.M. Rops^1^, Jo H. Berden^1^, Bart Smeets^3^, I. Jonathan Amster^2^, Ton J. Rabelink^4^, Johan van der Vlag^1^**

*** Correspondence:** Corresponding Author: [johan.vandervlag@radboudumc.nl](mailto:johan.vandervlag@radboudumc.nl)

# Supplementary Figure S1. mGEnC glycocalyx, mGEnC HSglx, mGEnC CSglx or enoxaparin do not affect induction of anti-GBM glomerulonephritis in mice. (a) Semi-quantitative analysis of rabbit anti-mouse GBM IgG in mouse glomeruli 2 hours, 1 day and 4 days after injection with anti-GBM IgG only, or anti-GBM IgG with mGEnC glycocalyx, mGEnC HSglx, mGEnC CSglx or enoxaparin. (b) Semi-quantitative analysis of C3c-deposition in glomeruli of mice 2 hours, 1 day and 4 days after injection with anti-GBM IgG only or anti-GBM IgG with mGEnC glycocalyx, mGEnC HSglx, mGEnC CSglx or enoxaparin. Staining intensities are expressed as means ±S.E.M. from 3-5 mice in arbitrary units (A.U.).

# Supplementary Figure S2. Immunofluorescence staining of fibrin in glomeruli of control and anti-GBM IgG injected mice. Representative immunofluorescence images for fibrin with anti- fibrinogen-fluorescein isothiocyanate(FITC) antibody (green) 4 days after injection with PBS, anti-GBM IgG and anti-GBM IgG + mGEnC-1 glycocalyx, HSglx, CSglx or enoxaparin.

# Supplementary Figure S3. Glomerular injury is not affected by mGEnC glycocalyx or enoxaparin in experimental anti-GBM glomerulonephritis. Semi-quantitative analysis of the percentage of affected glomeruli with thrombosis and hyalinosis within the glomerular capillaries, as analyzed by periodic Acid-Schiff (PAS) staining of de-paraffinized renal sections after 4 days anti-GBM nephritis. At least 50 glomeruli per mouse were analyzed. Results are expressed as means±S.E.M. from 3-5 mice per group in percentage of positive glomeruli.

# Supplementary Figure S4. Immunofluorescence staining of PMNs in glomeruli of control and anti-GBM IgG injected mice. Representative immunofluorescence images for PMNs with GR-1 antibody (green) and anti-agrin co-staining (red), 2 hours and 1 day after injection with PBS, anti-GBM IgG and anti-GBM IgG + mGEnC-1 glycocalyx, HSglx, CSglx or enoxaparin. White arrowheads indicate the presence of PMNs in the glomeruli.

# Supplementary Figure S5. Immunofluorescence staining of macrophages in glomeruli of control and anti-GBM IgG injected mice. Representative immunofluorescence images for macrophages with anti-CD68 antibody (green) and anti-agrin co-staining (red), 2 hours and 1 day after injection with PBS, anti-GBM IgG and anti-GBM IgG + mGEnC-1 glycocalyx, HSglx, CSglx or enoxaparin. White arrowheads indicate the presence of macrophages in the glomeruli.

# Supplementary Figure S6. HRGEC HSglx binds to recombinant human L-selectin and rhCD11b. HRGEC HSglx was incubated with 96-well plate-immobilized proteins and binding was probed withan anti-HS antibody. N= 5 replicates from two independent experiments.

# Supplementary Figure S7. mGEnC HSglx size exclusion chromatogram and fractionation. mGEnC HSglx was separated by size and collected in 1 mL fractions. Fractions were then pooled according to the subsequent elution time (minutes). AU, arbitrary units (absorbance, 210 nm). Disaccharides (dp2) elute within fraction 4. Full length HS (50-200 disaccharides) elutes in the first peak in fraction 1.

# Supplementary Figure S8. Capillary electrophoresis-mass spectrometry electropherogram of mGEnC HSglx F2. mGEnC HSglx was separated by capillary electrophoresis and the total abundance of ions measured by electrospray ionization mass spectrometry. Two dominant peaks were observed in the electropherogram at 16.23 and 19.83 minutes.

# Supplementary data File 1. Total Mass Spectrometry mass list. The raw mass data list from the mass spectrometer for mGEnC HSglx F2 after capillary electrophoresis-MS shown in Supplementary Figure S5.
